# Editorial: Mitochondrial therapy in neurological diseases

**DOI:** 10.3389/fnmol.2022.988792

**Published:** 2022-08-01

**Authors:** Jui-Chih Chang, Linyi Chen, Chuang-Rung Chang

**Affiliations:** ^1^Center of Regenerative Medicine and Tissue Repair, Institute of ATP, Changhua Christian Hospital, Changhua City, Taiwan; ^2^Department of Medical Science, National Tsing Hua University, Hsinchu, Taiwan; ^3^Institute of Molecular Medicine, National Tsing Hua University, Hsinchu, Taiwan; ^4^Institute of Biotechnology, National Tsing Hua University, Hsinchu, Taiwan

**Keywords:** mitochondrial dysfunction, neurological diseases, brain injury, metabolic disease, reactive oxygen species

Mitochondrial dysfunction has been implicated in mitochondrial encephalopathy, lactic acidosis and stroke-like episodes (MELAS), brain injury, and neurodegenerative diseases. Difficulty on mitochondrial gene-editing has been one of the major bottlenecks in correcting mitochondria-related disorders. To this end, mitochondrial transplantation presents a new paradigm of therapeutic intervention that benefits neuronal survival and regeneration for neurological diseases. This Research Topic focuses on the mechanistic evidence of mitochondrial dysfunction in neurological diseases and related metabolic disorder, current, and potential therapeutic approaches and supplements.

Mitochondrial function is susceptible to a delicate balance of fusion, fission, and degradation. Li et al. reviewed many faces of electron transport chain (ETC) complex individually affected by Parkinson's Disease (PD)-associated genes. As Figure 1 depicts, in PD, ETC complex regulates neural apoptotic death, mitochondrial crista structure stability, lysosome function, mitophagy, balance of membrane lipid, oxidative stress, and Lewy body formation, which are the key drivers of PD. Nonetheless, the role of mitochondrial dynamics in PD is rare mentioned largely due to lack the direct evidence. The elongated and uncharacteristically irregular morphology of mitochondria observed in knock-in mice with gene mutation of mitochondrial ubiquinol-cytochrome c reductase core protein 1, a subunit of complex III (Lin et al., 2020), links the association of PD-associated genes and ETC complexes on the direct regulation of mitochondrial morphogenesis.

**Figure 1:**
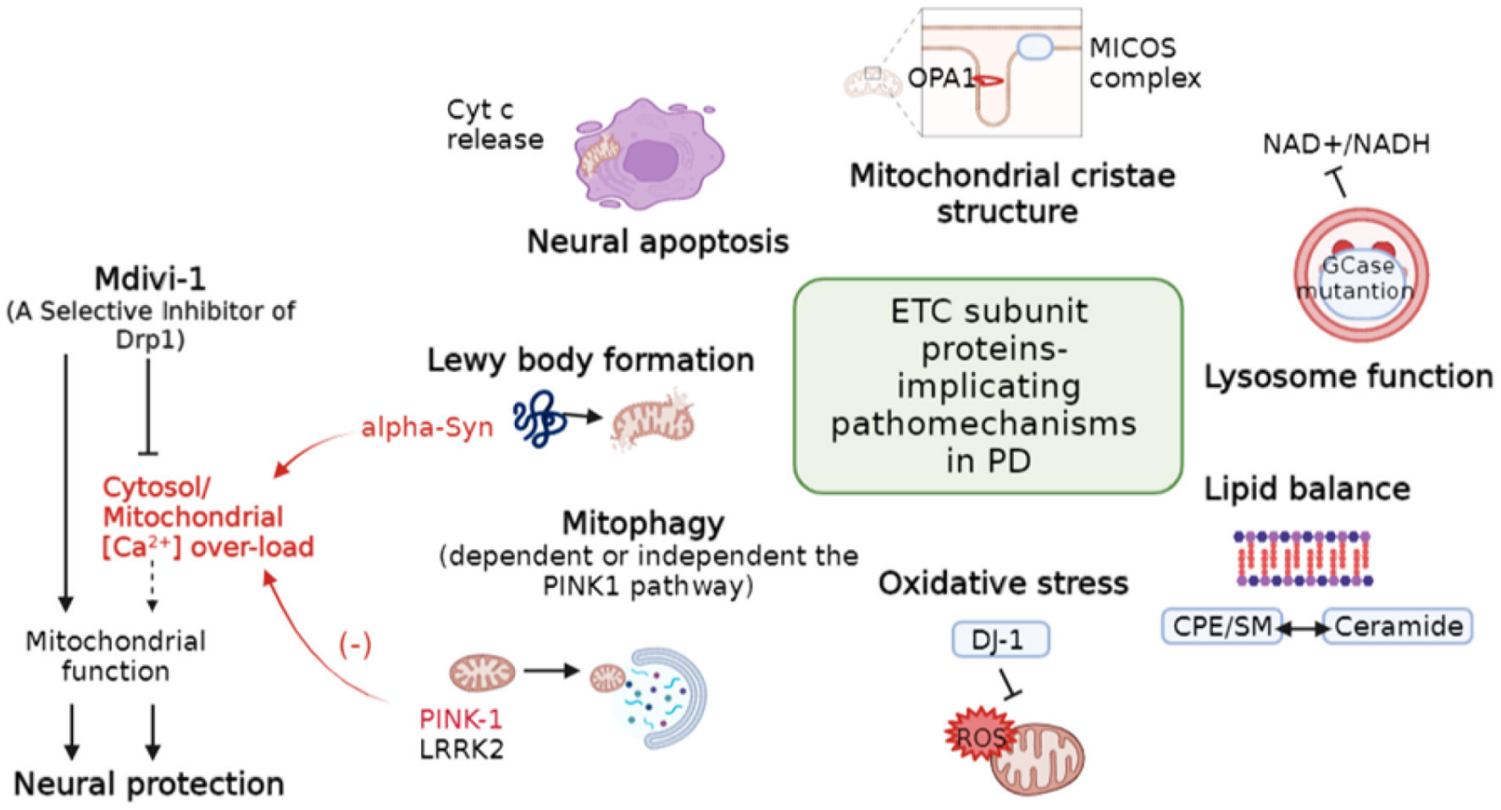
Diverse regulation of mitochondria-related pathomechanisms in PD. Dotted line indicates hypothetical links. PD, Parkinson's disease; Cytc, Cytochrome C; MICOS, Mitochondrial contact site and cristae organizing system; CPE, Ceramide phosphoethanolamine; SM, Sphingomyelin. (Modified based on the study: Li et al.).

On the other hand, PD-associated PINK-1 and Alpha-Synuclein (α-Syn) are involved in the balance of mitochondrial [Ca^2+^] (Figure 1). PINK-1 deficiency leads to a delayed calcium efflux and mitochondrial calcium overload upon physiological stimulation (Gandhi et al., 2012), whereas α-Syn increases cytosol calcium influx by forming a pore in the plasma membrane (Zakharov et al., 2007). Dysregulation of [Ca^2+^] is a major pathological hallmark not only for PD (Ludtmann and Abramov, 2018) but also for the other neurodegeneration diseases (Abeti and Abramov, 2015). Mitochondrial division inhibitor-1 (Mdivi-1) is a candidate neuroprotector against ischemia-reperfusion injury after stroke and the machinery is associated with support of mitochondrial function (Nhu et al.). While effect of Mdivi-1 remains controversial (Liu et al., 2015; Ruiz and Alberdi, 2018; Ruiz et al., 2020), its protective machinery in neurons is consistently reported to reduce [Ca^2+^] overload (Ruiz and Alberdi, 2018; Ruiz et al., 2020). Moreover, mitochondrial calcium uniporter stabilization preserves energetic homeostasis during Complex I impairment (Balderas et al., 2022). Thus, targeting calcium homeostasis could potentially provide therapeutic cues for neurological diseases.

As mitochondria-cytosol crosstalk buffers cellular ROS and [Ca^2+^], mitochondrial PGAM5 protein appears to initiate the action. Role of PGAM5 in mitochondrial morphogenesis and mitophagy have been documented in cancer cells. Its role in the nervous system is still unclear. A review by Liang et al. proposed a functional regulation by PGAM5 in promoting mitochondrial biogenesis and neuronal regeneration after traumatic brain injury (TBI). Stress-induced cleavage of PGAM5 traffics to cytosol, hijacks downstream effectors of WNT signaling and modulates transcription of mitochondrial biogenesis genes. This mechanism echoes a promising therapy of mitochondrial transplantation to partially rescue mitochondrial damage and promote neuronal regeneration.

When comparing the clinical outcome between men and women, women had better outcome after severe TBI. Kalimon and Sullivan nicely compared and discussed the known influence of estrogen, progesterone and testosterone on mitochondrial bioenergetics, glucose utilization at early/acute or later stages after TBI in both rodents and human patients. Estrogen and progesterone obviously take their unusual turn in partying with plasma membrane, mitochondria, and the nucleus. Maintaining cerebral spinal fluid (CSF) is a key for better outcome of TBI. In this aspect, TBI triggers high CSF in male rats whereas CSF in TBI females rats remains low. Glucose utilization and cerebral blood flow in hippocampus varies during different phases of the estrus cycle, suggesting a different susceptibility to glucose utilization and metabolism for males and females upon TBI. These evidence implicate a link between brain injury and metabolism.

Type 2 diabetes mellitus (T2DM) and PD had been demonstrated by a large cohort study that showed PD incidence increased in T2DM patients. Lin et al. reported the effects of an anti-diabetic drug, glucagon-like peptide-1 receptor agonists (GLP-1RAs), on mouse model of PD. Their results suggest possible shared genetic predisposition or pathogenic pathways for both diseases. The protective effect of GLP-1RAs in PD models was shown to involve the activation of GLP-1 receptors and downstream signaling. To this end, liraglutide, a GLP-1RA, was approved by U.S. Food and Drug Administration for treating diabetes, on 1-methyl-4-phenyl-1,2,3,6-tetrahydropyridine (MPTP)-induced PD mouse model. MPTP caused parkinsonism through its mitochondrial toxic on respiration complex I. Their results demonstrated that applying GLP-1RA did provide protection for mouse challenged by MPTP. It reduced the level of blood sugar reduction, protected dopaminergic neurons, and inhibited apoptosis in the substantia nigra caused by MPTP. GLP-1RA restored balance of mitochondria dynamics judged by the change of mitochondrial network from mega to fragmented form. The most significant result of this report is that GLP-1RA reduced α-Syn aggregation in substantia nigra, supporting a neuroprotective effect of the GLP-1RA and its potential as therapeutic agents that link type II diabetes and PD.

Almannai and El-Hattab reviewed the role of nitric oxide (NO) deficiency in mitochondria diseases. Deficiency of NO in mitochondria diseases has been implicated in Mitochondrial Encephalopathy, Lactic Acidosis, and Stroke-like episodes (MELAS). Since arginine and citrulline are substrates for NO synthesis, taking supplementation of arginine and citrulline could potentially aid patients with mitochondrial disorders resulting from NO deficiency, in particular for stroke-like episodes in patients with MELAS. Similar concept of identifying and characterizing supplements that may benefit mitochondria related neurological disorders is in conjugation with other published articles in this special topic collection.

## Author contributions

J-CC, LC, and C-RC wrote and revised the manuscript. Figure 1 was generated by J-CC. All authors contributed to the article and approved the submitted version.

## Funding

This work was supported by the Ministry of Science of Technology, Taiwan (grant#: MOST 111-2311-B-007-012).

## Conflict of interest

The authors declare that the research was conducted in the absence of any commercial or financial relationships that could be construed as a potential conflict of interest.

## Publisher's note

All claims expressed in this article are solely those of the authors and do not necessarily represent those of their affiliated organizations, or those of the publisher, the editors and the reviewers. Any product that may be evaluated in this article, or claim that may be made by its manufacturer, is not guaranteed or endorsed by the publisher.
